# Repurposing a commercial artificial intelligence vision platform for automated surgical instrument identification: a proof-of-concept study

**DOI:** 10.1186/s13037-026-00512-z

**Published:** 2026-10-12

**Authors:** Simon Stoerzer, Klinto Xhemo, Felix Gronau, Linda Feldbruegge, Philipp Felgendreff, Maximilian Kaltenecker, Jacques Hahn, Gerrit Grannas, Moritz Schmelzle

**Affiliations:** 1 Department of General, Visceral and Transplant Surgery, Hannover Medical School, Carl-Neuberg-Strasse 1, 30625 Hannover, Germany; 2 Nexustec GmbH, Munich, Germany

**Keywords:** Retained surgical items, Surgical count, Artificial intelligence, Computer vision, Surgical instrument recognition, Operating room, Human factors

## Abstract

**Background:**

Retained surgical items are classified as never events but continue to occur, and the count meant to prevent them is often recorded as correct even when an item is retained. Computer vision could provide an independent check on what is on the instrument table, but purpose-built surgical systems are scarce. Industrial vision platforms offer a faster route, although their surgical performance is unknown.

**Methods:**

In a prospective experimental study, a commercial artificial intelligence vision platform developed for industrial inspection was applied to 19 surgical instruments and 3 suture items, each presented once in 100 runs (2,200 experiments). No software redesign was required, but the classifier had been trained on this item set, making this a supervised rather than a zero-shot setting. Two outcomes were recorded: whether the platform assigned the exact item identity, and whether it registered an item as present at the packing step irrespective of type, termed count-presence detection. Because experiments are clustered within items and runs, the primary estimate is the mean per-item accuracy with cluster-adjusted intervals.

**Results:**

The correct instruments were identified in 2,036 of 2,200 experiments (92.5%). The mean per-item accuracy was 92.5% (95% confidence interval 88.4 to 96.7; cluster-adjusted 87.7 to 95.6). Count-presence detection was 99.1% (98.7 to 99.4). Performance was reproducible across runs (intraclass correlation 0.02), whereas variance clustered by item (0.12). Accuracy ranged from 64% (suture needle 6-0) to 100% (six items). Errors were directional and almost always within visually near-identical sub-types.

**Conclusions:**

An industrial vision platform detected item presence with high reliability and identified item type with high accuracy, however, with a residual error rate of 7.5%. The errors were predictable and confined to visually similar classes, pointing to correctable mitigations rather than a fundamental limit. These figures represent an upper bound under highly controlled conditions rather than expected intraoperative performance. Such a system is intended to support rather than replace the perioperative team. This study establishes feasibility rather than clinical benefit; a prospective intraoperative study is the necessary next step.

## Background

Surgical sponges, needles, and instruments left in a patient after wound closure, collectively termed retained surgical items, are among the most avoidable serious complications in operative practice and are classified as never events [1, 2]. They have not been eliminated. Depending on the case mix and detection method, a retained foreign object is reported in roughly one in several thousand operations, with higher rates in body-cavity and emergency procedures [1, 2]. Sharps deserve particular attention, since needles, blades, wires and instrument fragments account for a rising share of reported events [3]. The harm extends beyond the physical injury to reoperation, litigation, and loss of trust, and the cost of a single event runs into tens of thousands of dollars [4, 5].

The manual surgical count is the main safeguard, yet it is a fragile one. In a large share of confirmed retention cases the final count had been recorded as correct, so the process meant to catch the error had already failed by the time the item was found [1, 2]. Count discrepancies are common in routine practice and take time and attention to resolve [6], and formal analysis has questioned how much protection counting alone provides [7]. The count is also carried out in a demanding environment, under time pressure and frequent interruption, all of which can degrade accuracy [6]. Instrument labeling and tray layout have themselves been proposed as contributing factors [8]. The World Health Organization surgical safety checklist and adjunct technologies such as radiofrequency tagging and barcoded sponges were introduced precisely because human vigilance on its own is not enough [5, 9–11].

Computer vision is an attractive complement to the count because it can provide a continuous and independent record of what is on the table. Deep learning has advanced surgical video analysis quickly, from early networks for laparoscopic tool-presence detection to instrument detection, segmentation, and tracking [12–14], and machine learning is increasingly discussed as a general instrument for improving perioperative safety [15, 16]. Recognition accuracies commonly sit in the low-90% range for comparable tasks, and the approach has been extended to robotic scrub-nurse assistance [17, 18], to real-time tracking in laparoscopy [19], to cholecystectomy video supported by synthetic image generation [20], to endoscopic skull base surgery [21], and to reading the identification codes etched on instruments [22]. Closest to the present question, a model trained specifically for surgical tool detection and counting reached precision of 94.0% to 100% and recall of 97.1% to 100% across eleven categories [23]. Progress is therefore real but uneven, and systems validated specifically to support the instrument count remain rare.

One pragmatic route is to reuse vision technology that is already mature in other fields. Automated optical inspection in manufacturing has solved high-throughput, fine-grained visual classification for years, often exceeding 95% accuracy under controlled imaging, although robustness outside controlled conditions remains the main obstacle to deployment [24]. Whether such a platform transfers to the cluttered and safety-critical surgical setting is untested.

We therefore evaluated a commercially available artificial intelligence vision platform originally developed for automotive and industrial inspection. The platform was applied to a complete standardized set of surgical instruments and suture items without any task-specific redesign of the software, although its classifier was trained on this item set beforehand, so the evaluation was carried out in a supervised rather than a zero-shot setting. Our aim was to assess its capabilities and limitations in identifying these items and to determine the adaptations required for its potential use in surgical counting.

## Methods

### Study design

This was a prospective, controlled, experimental study of the recognition performance of a commercial device. It involved no patients, no human tissue, and no animals, and no identifiable data were collected. There was no clinical comparison tool. The study was designed to quantify recognition and error structure under controlled conditions rather than to measure a clinical outcome. Reporting follows the Checklist for Artificial Intelligence in Medical Imaging (CLAIM) and its 2024 update [25, 26]. Because the present work is an experimental study without patient involvement, the DECIDE-AI early-stage clinical evaluation guideline [27] is cited as the framework for the planned intraoperative study rather than for this one.

### Recognition platform

The evaluated system was a commercial artificial intelligence vision and automation platform (TRiMiTi, Nexustec^®^, Munich, Germany) whose prior use has been in automotive and industrial inspection rather than healthcare. It performs camera-based image acquisition and classifies each presented object against a predefined catalog of registered instrument classes under vendor-supplied configuration. The internal architecture, training data, and any per-class confidence outputs are proprietary and were not disclosed to us, so we treated the platform as a black box and report only its externally observable behavior. The supplier had no role in the design, conduct, analysis, or interpretation of this study. The relationship between the items evaluated here and the data used to configure the platform is central to interpreting our results, so we state it explicitly. Two things must be distinguished. The software itself was used as delivered and was not redesigned, re-engineered, or otherwise adapted for the surgical task. The classifier, however, was not applied zero-shot: the 22 items were registered into the platform catalog and images of this set were used to train the model weights before the runs began. The evaluation therefore measures supervised performance on the configured catalog rather than generalization to previously unseen items, a distinction we return to in the Limitations.

### Image acquisition and presentation protocol

Items were presented individually to the platform camera under standardized conditions. Images were acquired at a working distance of approximately 30 cm, against a plain white background. All items were presented clean and dry. Hinged instruments, that is scissors, clamps, and needle holders, were presented in both the closed and the open position, and the double-armed 6 − 0 suture was presented with both of its needles visible. Items were presented in randomized order, and the orientation of each item was freely chosen rather than fixed. The setup and the outcome definitions were held constant across all 100 runs, so that between-run variation reflects platform behavior rather than changes in the presentation protocol.

### Item set

The test set comprised 22 items: 19 surgical instruments covering the main functional classes of a general tray, namely grasping and holding forceps, hemostatic and towel clamps, needle holders, scissors, retractors and hooks, and a sponge-holding grasper, together with 3 suture items (Table 1). Suture material was included deliberately, because needles are counted alongside instruments and are the item most often miscounted in clinical practice [3], and because a fine needle poses a harder recognition problem than a solid instrument. For brevity we refer to all 22 as items, and use instrument for the 19 non-suture items. The set deliberately included several near-duplicate pairs, such as fine versus coarse surgical forceps, anatomical versus toothed surgical forceps, short versus long needle holders, and fine versus coarse utility scissors, so that fine-grained discrimination could be tested.

**Table 1:** The 22-item validation set, comprising 19 surgical instruments and 3 suture items, grouped by functional class. The set deliberately includes several near-duplicate pairs to test fine-grained discrimination

#	Item	Functional class
1	Mosquito clamp	Clamp
2	Péan clamp	Clamp
3	Splinter forceps	Forceps
4	Utility scissors, coarse	Scissors
5	Surgical forceps, fine	Forceps
6	Anatomical forceps	Forceps
7	Anatomical forceps, fine	Forceps
8	Surgical forceps, coarse	Forceps
9	Fine dissecting clamp	Clamp
10	Lid retractor	Retractor/Hook
11	Backhaus towel clamp	Clamp
12	Microsurgical forceps	Forceps
13	Sponge-holding forceps	Grasper
14	Needle holder, short	Needle holder
15	Utility scissors	Scissors
16	Self-retaining retractor	Retractor/Hook
17	Sharp retractor hook	Retractor/Hook
18	Needle holder, long	Needle holder
19	Vein retractor	Retractor/Hook
20	Suture needle 2 − 0	Needle/Suture
21	Suture needle 6 − 0	Needle/Suture
22	Double-armed suture 6 − 0	Needle/Suture

### Endpoints and outcome adjudication

**Fig. 1:**
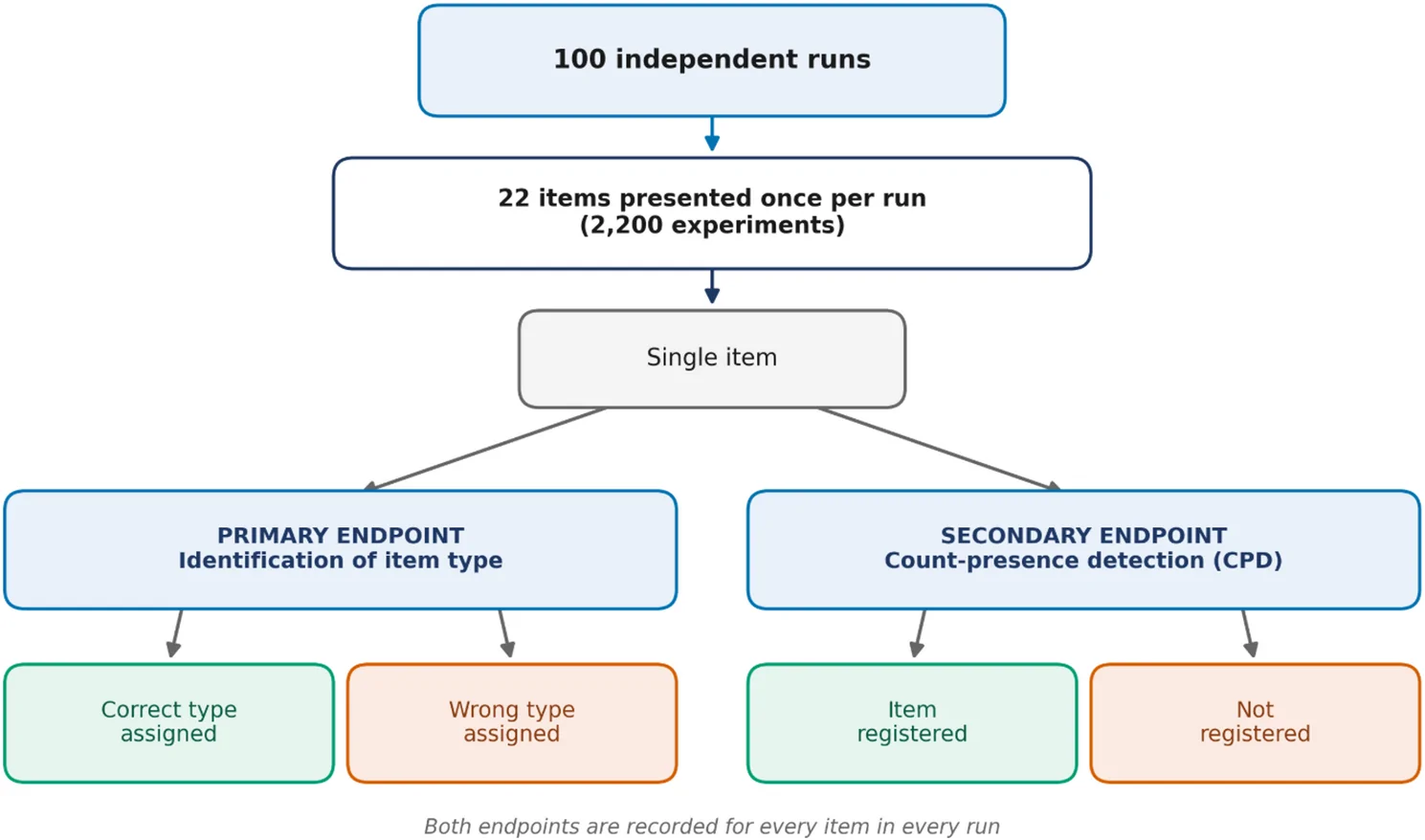
Study workflow and endpoint recording. Each of 2,200 experiments was recorded independently on two endpoints: identification (correct item type versus an incorrect label) and count-presence detection (registered versus not registered at the packing step)

One hundred independent runs were performed. In each run, all 22 items were presented once, giving 2,200 experiments. Two outcomes were recorded per experiment (Fig. 1). Each run followed the same sequence. The 22 items were presented to the camera one after another, and the label returned by the platform was recorded for each of them. Every item was then placed into the tray, and the platform recorded whether it registered the item at this packing step, so that both endpoints were obtained for every item within the same run. The primary endpoint was correct identification according to the exact catalog identity of the presented item, recorded as correct or incorrect. For incorrect calls, the erroneously assigned label was recorded to allow confusion analysis. The secondary endpoint, which we term count-presence detection (CPD), recorded whether the platform registered that an item was present, irrespective of which item it was. CPD is not an established term in the literature; we use it here to name the presence-or-absence task that underlies the surgical count. It was recorded at the packing step as detected or not detected. The two endpoints capture different tasks: exact identity, which matters for automated inventory, and mere presence, which is the quantity most relevant to preventing a retained item. In two experiments the platform assigned a label that lay outside the 22-item registered catalog; these out-of-catalog calls were scored conservatively as misclassifications. Outcomes were recorded live during each run.

### Statistical analysis

Because every item was presented exactly once in each run, the design is balanced, and the mean per-item accuracy therefore yields the same point estimate as the pooled proportion. The two differ not in the estimate itself but in how uncertainty around it is quantified, which is why we report both. The 2,200 experiments are not independent, since the same 22 physical items were presented repeatedly and the observations are clustered within items and within runs; an interval that assumes independence therefore understates the true uncertainty. Our primary estimate is the mean per-item accuracy across the 22 items, that is the mean of the individual item accuracies, which weights every item equally, with a 95% confidence interval derived from the between-item variability using the t distribution on 21 degrees of freedom. This treats the item as the unit of generalization. As a second estimate we fitted a generalized estimating equation with an exchangeable working correlation clustered by item, which yields a cluster-adjusted interval, that is an interval widened to account for the repeated measurement of the same items. For reference we also give the pooled proportion with a Wilson score interval, which assumes independence and is therefore optimistic. Reproducibility across runs and clustering by item are summarized with intraclass correlation coefficients from one-way random-effects models. Simple proportions elsewhere use Wilson intervals [28]. Analyses used Python (pandas, statsmodels) with figures in Matplotlib. A large language model was used for language and grammar editing of the manuscript. The authors reviewed and take full responsibility for the content.

## Results

The platform assigned the correct item identity in 2,036 of 2,200 experiments. Accounting for clustering, the mean per-item accuracy across the 22 items was 92.5% (95% CI 88.4 to 96.7), with a between-item standard deviation of 9.4% points; the cluster-adjusted estimate agreed closely (95% CI 87.7 to 95.6). Because the design is balanced, the pooled proportion is numerically identical at 92.5%, but its Wilson interval (91.4 to 93.6) assumes independence, is about a quarter as wide, and therefore overstates the precision. Count-presence detection was higher and close to the ceiling at 99.1% (2,181 of 2,200; 95% CI 98.7 to 99.4). The gap between the two endpoints is the central finding: the platform was more reliable at confirming that an item was present than at naming which item it was.

### Reproducibility and clustering

**Fig. 2:**
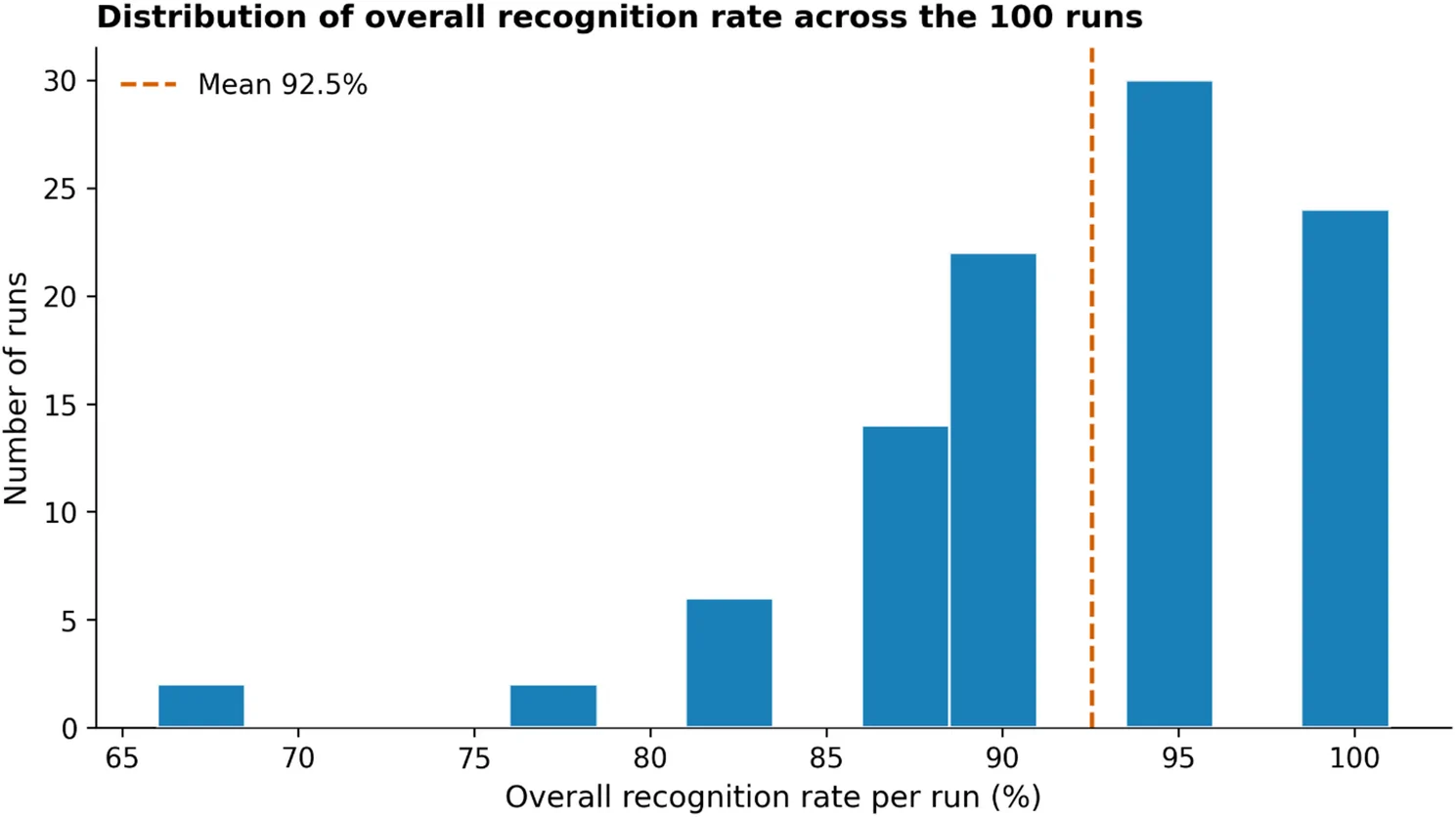
Distribution of the overall recognition rate across the 100 runs. Most runs sit near the ceiling; the tail reflects the repeated failure of a few near-duplicate items rather than random error

Performance was stable from run to run. The run-level intraclass correlation was 0.02, indicating that very little of the variance sat between runs and that the platform behaved consistently on repetition. The per-run recognition rate averaged 92.5% (standard deviation 6.8% points; range 68.2% to 100%), and 24 of the 100 runs were error-free (Fig. 2). By contrast, variance clustered by item (item-level ICC 0.12), confirming that errors were driven by which item was shown rather than by random run-to-run noise.

### Per-item performance

**Fig. 3:**
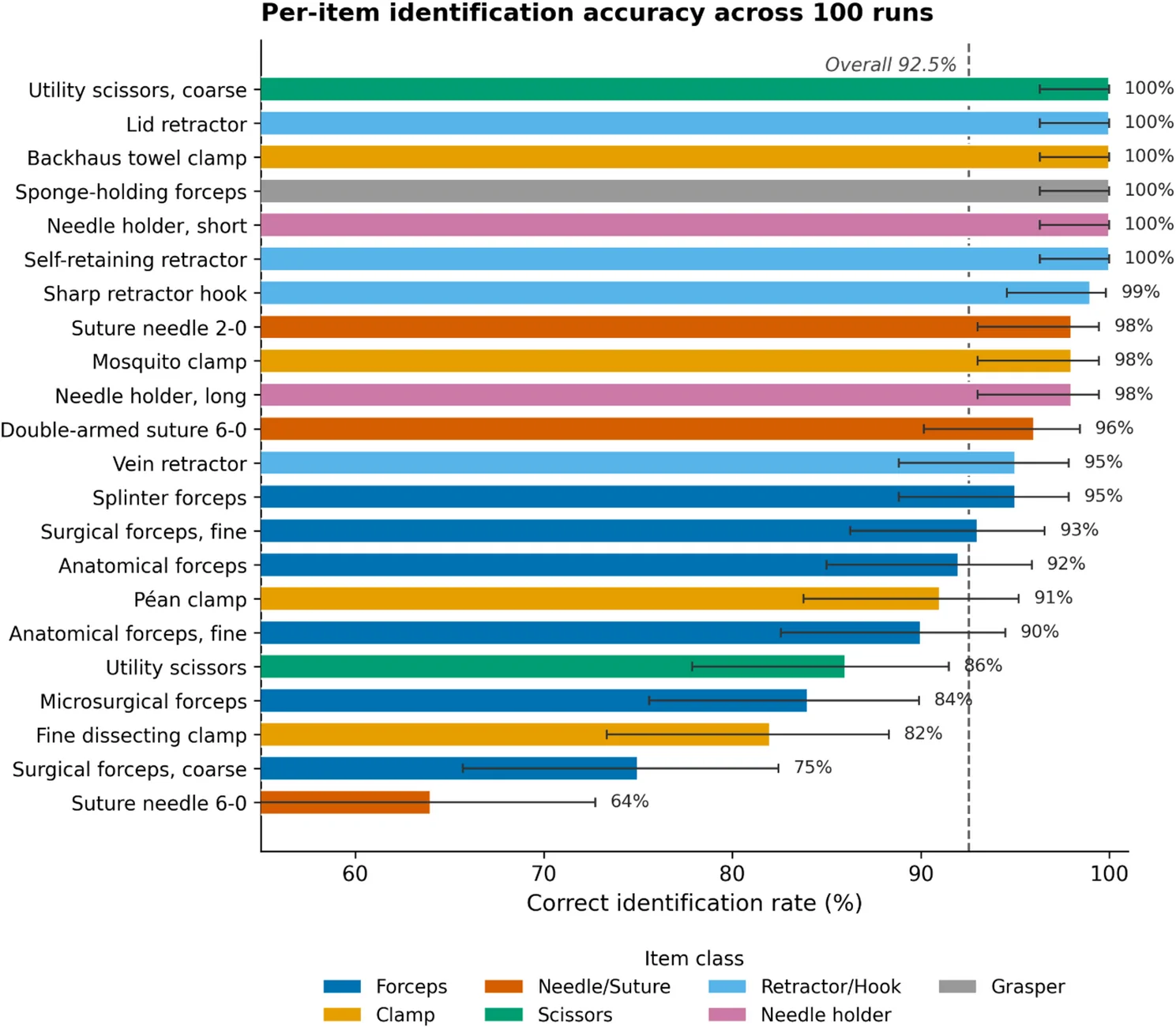
Per-item identification accuracy across 100 runs, sorted ascending and colored by item class. Error bars are 95% Wilson score intervals; the dashed line marks the pooled accuracy. Forceps and fine sutures dominate the low-accuracy end

Accuracy varied widely by item, from 64% to 100% (Fig. 3; Table 2). Six items were classified correctly in every run: the self-retaining retractor, short needle holder, sponge-holding forceps, Backhaus towel clamp, lid retractor, and coarse utility scissors. The lower tail was driven by five items: the 6 − 0 suture needle (64%), the coarse surgical forceps (75%), the fine dissecting clamp (82%), the microsurgical forceps (84%), and the fine utility scissors (86%). Every item in this tail belongs to a visually near-identical pair or family.

**Table 2:** Per-item recognition results across 100 runs, ordered by ascending accuracy. Count-presence is the packing-step detection rate; the final column gives the most frequent erroneous label

Item	Runs	Correct	Rate %	95% CI	Count-pres.	Dominant misclassification (*n*)
Suture needle 6 − 0	100	64	64	54.2–72.7	98/100	Double-armed suture 6 − 0 (*n* = 28)
Surgical forceps, coarse	100	75	75	65.7–82.5	100/100	Anatomical forceps (*n* = 21)
Fine dissecting clamp	100	82	82	73.3–88.3	98/100	Mosquito clamp (*n* = 11)
Microsurgical forceps	100	84	84	75.6–89.9	100/100	Anatomical forceps (*n* = 8)
Utility scissors	100	86	86	77.9–91.5	97/100	Utility scissors, coarse (*n* = 14)
Anatomical forceps, fine	100	90	90	82.6–94.5	100/100	Anatomical forceps (*n* = 6)
Péan clamp	100	91	91	83.8–95.2	99/100	Sponge-holding forceps (*n* = 5)
Anatomical forceps	100	92	92	85.0–95.9	100/100	Anatomical forceps, fine (*n* = 4)
Surgical forceps, fine	100	93	93	86.3–96.6	100/100	Anatomical forceps (*n* = 6)
Splinter forceps	100	95	95	88.8–97.8	100/100	Microsurgical forceps (*n* = 2)
Vein retractor	100	95	95	88.8–97.8	99/100	Sharp retractor hook (*n* = 3)
Double-armed suture 6 − 0	100	96	96	90.2–98.4	98/100	Suture needle 6 − 0 (*n* = 3)
Mosquito clamp	100	98	98	93.0–99.4	100/100	Needle holder, short (*n* = 1)
Needle holder, long	100	98	98	93.0–99.4	99/100	Sponge-holding forceps (*n* = 1)
Suture needle 2 − 0	100	98	98	93.0–99.4	99/100	Microsurgical forceps (*n* = 1)
Sharp retractor hook	100	99	99	94.6–99.8	100/100	Vein retractor (*n* = 1)
Utility scissors, coarse	100	100	100	96.3–100.0	99/100	None
Lid retractor	100	100	100	96.3–100.0	99/100	None
Backhaus towel clamp	100	100	100	96.3–100.0	98/100	None
Sponge-holding forceps	100	100	100	96.3–100.0	98/100	None
Needle holder, short	100	100	100	96.3–100.0	100/100	None
Self-retaining retractor	100	100	100	96.3–100.0	100/100	None

### Performance by item class

**Fig. 4:**
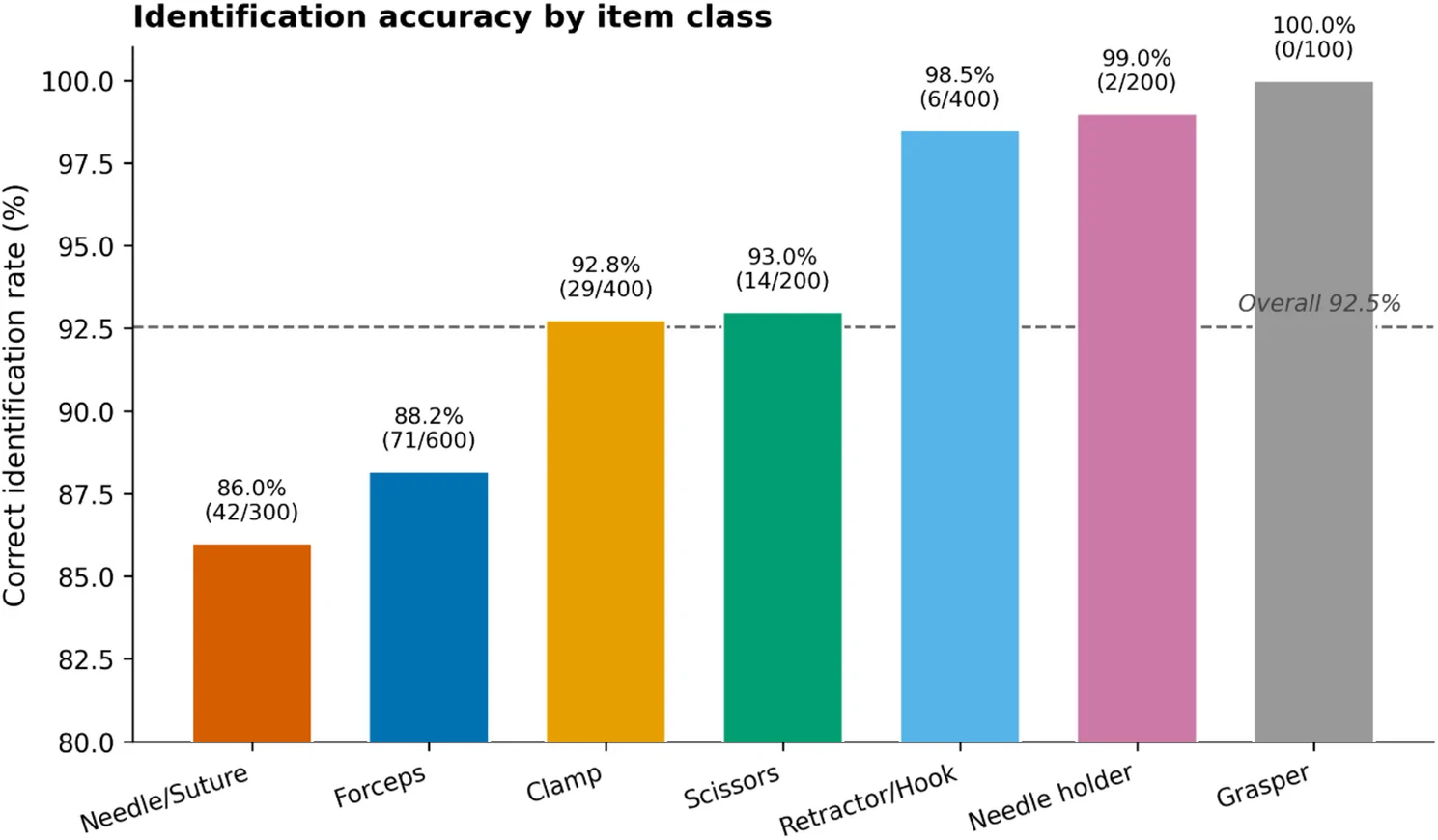
Identification accuracy by item class. The forceps and needle/suture classes, which are richest in near-duplicate sub-types, account for most of the errors

Aggregating by class made the pattern clear (Fig. 4; Table 3). The sponge-holding grasper (100%), needle holders (99.0%), and retractors or hooks (98.5%) were essentially solved. Scissors (93.0%) and clamps (92.8%) sat near the pooled mean. The two weakest classes were forceps (88.2%) and needles or sutures (86.0%), which are the categories that contain the largest number of fine-grained near-duplicate sub-types.

**Table 3:** Recognition accuracy aggregated by item class, ordered by descending accuracy

Functional class	Experiments	Correct	Errors	Rate %
Grasper	100	100	0	100.0
Needle holder	200	198	2	99.0
Retractor/Hook	400	394	6	98.5
Scissors	200	186	14	93.0
Clamp	400	371	29	92.8
Forceps	600	529	71	88.2
Needle/Suture	300	258	42	86.0

### Structure of the errors

**Fig. 5:**
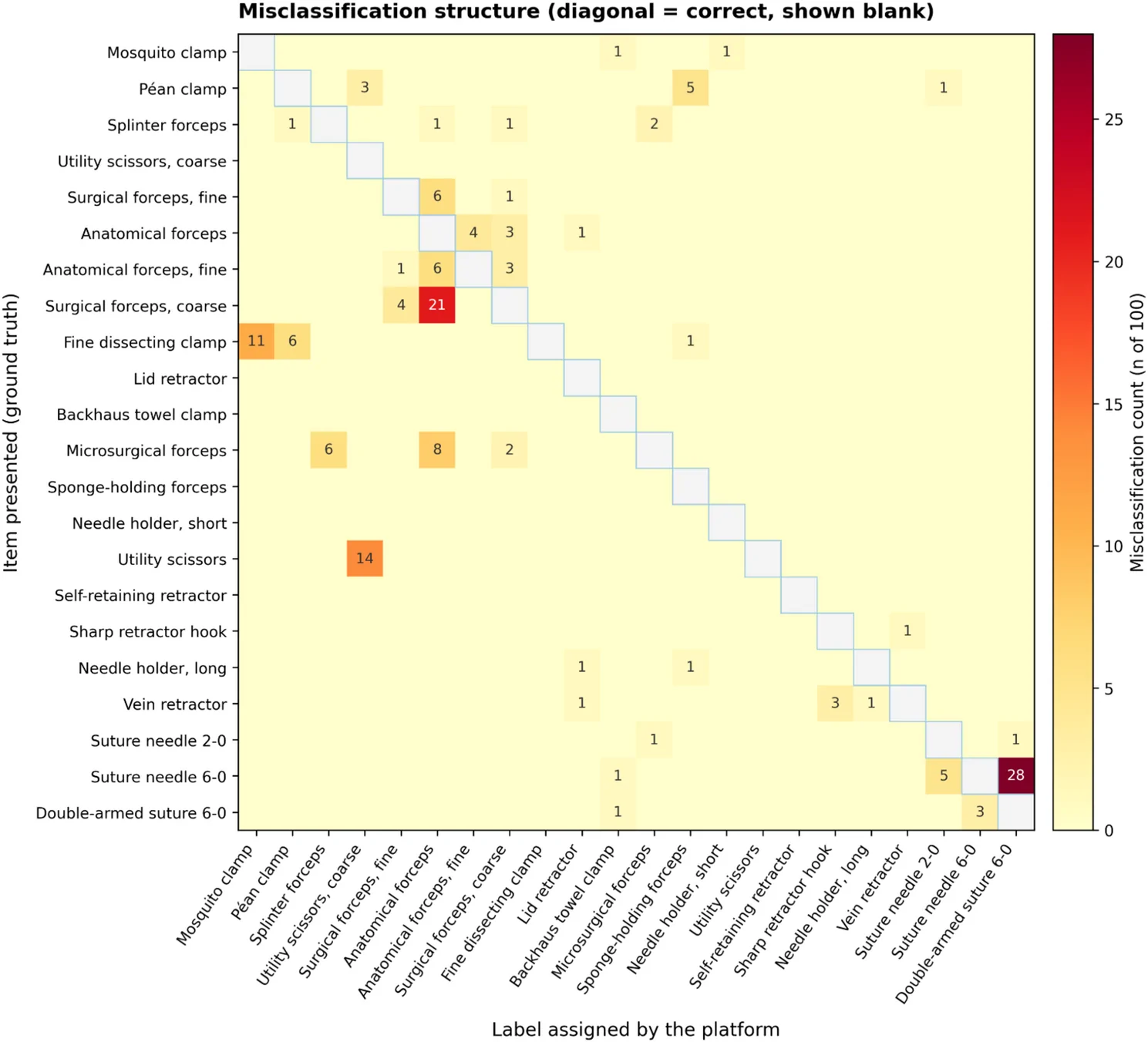
Misclassification structure across all 2,200 experiments. Rows are the item presented; columns are the label the platform assigned. Correct calls (the diagonal) are omitted so that the off-diagonal error channels stand out. Errors concentrate in compact blocks within the forceps, clamp, and suture families

**Fig. 6:**
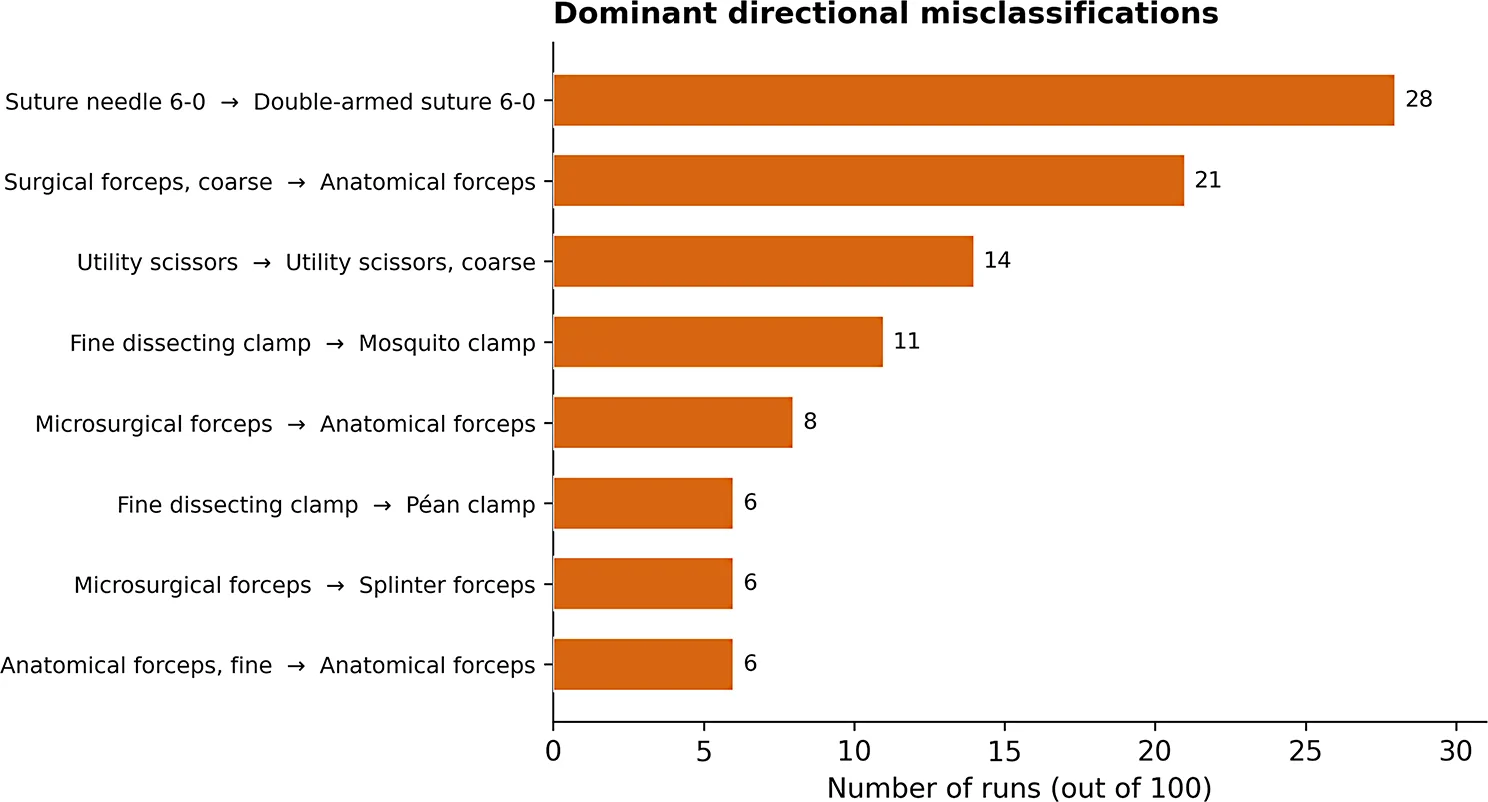
The eight most frequent directional misclassifications. Each bar counts the runs (out of 100) in which the item on the left was labeled as the item on the right. Every dominant confusion is within a class

Misclassifications were not spread evenly. They formed a small number of strong, directional channels between visually similar items (Figs. 5 and 6; Table 4). Three channels dominated. The 6 − 0 suture needle was labeled as a double-armed 6 − 0 suture in 28 of 100 runs, a confusion between an item and its double-armed variant. The coarse surgical (toothed) forceps was labeled as an anatomical (non-toothed) forceps in 21 runs. The fine utility scissors were labeled as their coarse counterpart in all 14 of their error runs, a one-directional confusion of scale. Further recurrent errors occurred within the forceps family, where microsurgical, anatomical, splinter, and surgical forceps were interchanged in both directions, and among the small clamps, where the fine dissecting clamp was taken for a mosquito or Péan clamp.

These channels matched failure modes that the operating team had anticipated from item geometry before the runs began. A toothed surgical forceps seen from above shows almost the same flat outline as a non-toothed anatomical forceps. A single-armed 6 − 0 needle is hard to separate from its double-armed version. Fine dissecting clamps need to be shown open, or with the tips at ninety degrees to the camera, or they read as a Péan clamp. Microsurgical and splinter forceps seen side-on in the open position collapse onto the anatomical-forceps class. Each of these predictions held in the data, which indicates that the errors come from viewing-angle dependence and genuine visual similarity rather than from noisy labeling.

**Table 4:** The eight most frequent directional misclassifications (presented to assigned label), counted over 100 runs

Presented → assigned label	Runs (of 100)
Suture needle 6 − 0 → Double-armed suture 6 − 0	28
Surgical forceps, coarse → Anatomical forceps	21
Utility scissors → Utility scissors, coarse	14
Fine dissecting clamp → Mosquito clamp	11
Microsurgical forceps → Anatomical forceps	8
Fine dissecting clamp → Péan clamp	6
Microsurgical forceps → Splinter forceps	6
Anatomical forceps, fine → Anatomical forceps	6

## Discussion

In an experimental study comprising 2,200 experiments, a vision platform built for industrial inspection identified surgical instruments and suture material with a mean per-item accuracy of 92.5% and detected item presence in 99.1% of cases. Its errors were few, strongly directional, and almost always confined to visually near-identical sub-types. The platform is therefore already close to sufficient for the task on which the count depends, and its remaining weakness lies in a different and more tractable task.

### Presence and identity are two different tasks

Separating count-presence detection from identification is the key to reading these results. Preventing a retained item is at heart a presence-or-absence problem. The safety question is whether every item that entered the field has left it, not whether its exact sub-type can be named. On that presence task the platform reached 99.1%. Most identity errors, by contrast, were swaps within a class, one forceps for another or a needle for its double-armed twin, which do not change the total number of items on the table. A system used to reconcile counts would therefore be more dependable than the raw identification rate suggests. The needle is the one exception that deserves attention, because needles and other sharps are among the most frequently miscounted items and account for a growing share of retained-item events [1, 3, 6], so the 6 − 0 confusion, although within a class, is the error we would least want to carry forward.

This limitation should be solvable. In a single view the two sutures look almost alike, and what separates them is the presence of a second needle. Both needles were visible during presentation, so the information needed to tell the two apart was contained in the image, and the platform simply did not weight it strongly enough. Errors of that kind respond to training rather than to hardware, and more examples of these specific pairs, shown across a wider range of angles and distances, would be expected to correct them. Synthetic generation of additional views, used successfully for instrument recognition in laparoscopic video [20], offers one way to obtain them. The pattern in our data supports this, because the errors were reproducible and ran in one direction only, which is what a slightly misplaced decision boundary looks like rather than a genuinely ambiguous task. Acquisition can help as well, and a second or third camera viewing the item from a different angle would make the distinguishing feature harder to miss, but the primary remedy here is training. A model trained on surgical instruments instead of an industrial catalog, combined with an improved viewing geometry, should therefore recover much of the lost accuracy.

### From the factory floor to the operating room

The main contribution here is speed of translation rather than a new algorithm. Instead of building a surgical dataset and training a model from scratch, which is the resource-intensive path most surgical-vision projects take, we took a platform already hardened by industrial inspection, left its software untouched, and trained it on a surgical tray. That it reached near-ceiling presence detection on first contact suggests that mature vision technology from other industries can be redirected into surgery in a useful time frame. The relevance is easy to state. The manual count is the last barrier against retained items, yet it depends on human performance under precisely the conditions that undermine it, namely time pressure, interruption, fatigue, and divided attention, and it fails silently in a meaningful share of the cases it is meant to catch [1, 2], so an automated and staff-independent check adds redundancy where human attention is known to lapse. Perioperative teams increasingly work short-staffed and under heavy cognitive load, and counting suffers under exactly those conditions [6], so a tireless check is most useful when the team is most stretched. Reliable instrument recognition is also a building block of surgical data science, feeding not only the count but also inventory, sterilization tracking, and documentation [29, 30]. Seen this way, the study is an early and concrete example of importing proven engineering into surgical care.

### Mechanisms behind the errors and how they can be corrected

The match between the anticipated geometric failure modes and the measured confusions points to specific, addressable causes. Four recur: close visual similarity between sub-types; dependence on viewing angle, since a toothed forceps loses its defining feature when seen from above; ambiguity of scale between otherwise identical fine and coarse variants; and a catalog granularity that forces the model to separate classes that a single static image barely distinguishes. Each has a practical mitigation, and none requires abandoning the platform:

- Standardize the acquisition geometry. A fixed presentation orientation, or several views per item, for example two or more cameras at different angles and distances, would restore the discriminating features, such as teeth, jaw profile, and needle arming, that a single top-down image hides.
- Adopt a hierarchical, count-oriented label scheme. Classifying to functional class first, and to exact sub-type second, guarantees that a needle is never counted as a forceps even when the sub-type is uncertain, which aligns the output with the count that safety depends on.
- Apply class-aware confidence thresholds with a human in the loop. Low-confidence calls, which are concentrated in a handful of known pairs, can be routed for a one-touch confirmation rather than accepted silently.
- Fine-tune on the confusable pairs. Targeted augmentation and retraining on the specific channels found here, 6 − 0 versus double-armed suture, toothed versus non-toothed forceps, and fine versus coarse scissors, address the errors where they occur.
- Design the catalog around count-equivalence. Where two sub-types are interchangeable for counting, merging them removes an error class at no cost to safety.

### Relation to prior work

The accuracy we observed is in line with the surgical-vision literature, where recognition tasks have commonly clustered in the low-90% range and tightly constrained, purpose-built modules have reached higher [13, 17]. The most instructive comparison is with a purpose-built model. Deol and colleagues trained a detector on 1,004 surgical images containing 13,213 tools across eleven categories and reported precision of 94.0% to 100% and recall of 97.1% to 100% [23]. Two differences matter. Their categories were broad and visually distinct, whereas ours were deliberately loaded with near-duplicate sub-types separated by a single feature; and their model was purpose-built, whereas ours was a generic industrial classifier trained on a surgical catalog. Our residual 7.5% therefore plausibly reflects architecture and catalog granularity rather than a limit of automated identification, which makes a direct comparison under identical conditions the obvious next experiment. Our results also mirror the platform’s industrial origin, where inspection systems perform strongly under controlled imaging but lose ground when conditions become variable [24]. They are a surgical instance of that same gap: strong performance under controlled conditions, with residual errors that expose the fine-grained, viewing-dependent brittleness separating an experimental result from a deployable tool. Because inadequate validation is a recognized reason that clinical AI fails to translate [27, 30], we have deliberately foregrounded the error structure and the clustered analysis.

### Beyond the count

Identity is the simplest question instrument recognition can answer, and for the count it is sufficient. What matters clinically is whether an instrument was used, when, in what order, and how well, rather than which manufacturer supplied it, and intraoperative tracking has begun to move in that direction [19, 21]. Our results are a prerequisite for that agenda rather than a contribution to it, since a system that cannot separate a fine forceps from a coarse one cannot credibly assess how either was used. The setting in which such data are most needed is also the one in which they are scarcest: robotic and endoscopic procedures generate video as a by-product of how they are performed, and the field’s benchmark datasets reflect this [12, 20], whereas open surgery, still the majority of operations worldwide, generates almost none. The instrument table, by contrast, is present in every operation and can be observed without altering it. Realizing that potential requires more than a classifier: time-stamped logs in a standardized vocabulary, for which machine-readable instrument codes offer one route [22], and prospective linkage to outcomes, since relating instrument use to results demands longitudinal records that remain rare for any surgical approach.

### Limitations

Several features of the design limit what can be concluded. First, and most important for how these figures should be read, conditions were far more favorable than an operating room. Each item was presented alone, clean, dry and unobscured against a plain background under constant light. A live field adds blood and irrigation fluid, drapes and swabs, overlap on a crowded stand, oblique angles, motion, and specular light, and the combined effect of these cannot be estimated from our data. Even a purpose-built surgical detector lost precision on images containing overlapping tools [23], and loss of robustness outside controlled imaging is the main reason industrial vision systems fail to reach deployment [24]. The 92.5% and 99.1% reported here are therefore an upper bound, not an estimate of intraoperative performance, and quantifying that gap is the most important next step. Second, the classifier had been trained on the same 22 items that were then evaluated, so this is a supervised internal validation on the configured catalog rather than a test of generalization. Our figures describe how well the platform recognizes items it has already learned, and say nothing about how it would perform on an unfamiliar tray, on instruments from another manufacturer, or at another site, all of which remain untested. The effective sample for generalizing across item types is the 22 clusters, which is why the clustered interval is wide. A zero-shot evaluation on an unseen item set, and a retraining and testing cycle on a separate set, are both natural next experiments. A third, equally important, is a head-to-head evaluation under identical acquisition conditions of this platform against a model trained specifically for surgical identification, which alone can separate the contribution of the generic architecture from that of the task. The platform was also evaluated as a vendor-configured black box, so the results describe this configuration rather than the underlying model. Third, the design has no comparator. Without a concurrent manual count or a second system as reference we can establish feasibility and map failure modes, but we cannot yet quantify any benefit over current practice. Fourth, the two endpoints are operational definitions tied to this platform’s workflow, and two calls fell outside the registered catalog and were scored as errors. We also did not record the number of needles as a separate endpoint, although the needle count is clinically the most critical single quantity in the surgical count, and a dedicated needle-count endpoint should form part of any prospective evaluation.

None of this overturns the two central observations, near-ceiling presence detection and a systematic, geometry-driven error structure, but together they set the next study: a prospective evaluation of the scrub nurse working with the system, compared with the nurse working alone, under realistic conditions, with independent ground-truth adjudication and, ideally, more than one item set and site. That study should be reported against the DECIDE-AI guideline [27]. That evaluation should be staged, and the central sterile supply department is the more feasible place to begin. Items are handled individually during tray assembly, lighting and background are controlled, there is no blood, no drapes, and no time pressure, and an incorrect label can be corrected without any risk to a patient. It is also the setting to which our results transfer most directly, because the acquisition conditions resemble those of the present study. There the same system could check a tray twice, on arrival and again after reprocessing before it is sealed, which is where a missing instrument is otherwise noticed late or not at all. Once the interplay of staff and system has been characterized there, the same design can be carried into the operating room, where conditions are harder but the potential safety benefit is greatest. In either setting the design should follow the way the system is meant to be used. We do not propose to test the platform against the scrub nurse, but to test the nurse supported by the platform against the nurse working alone. Responsibility for the count stays with the perioperative team, and the system adds a continuous and fatigue-free record that can flag a discrepancy for the team to resolve. Suitable endpoints are the detection of count discrepancies, the time needed to resolve them, and the workload and acceptance reported by the staff.

## Conclusions

A commercial vision platform taken from industrial inspection, used without any redesign of its software and trained on the item set, detected the presence of surgical instruments and suture material with high reliability and identified their exact type with high accuracy, at a residual error rate of 7.5%. The errors fell within a small, predictable set of visually near-identical sub-types that maps onto known and correctable causes. For supporting the count and reducing retained items, the safety-critical presence task is close to solved, and the identification task can be brought toward clinical standard through standardized viewing geometry, hierarchical labeling, targeted fine-tuning, and human confirmation of low-confidence pairs. These figures were obtained under highly controlled conditions and define an upper bound rather than expected performance in a live field. The study also shows how quickly mature technology from outside medicine can be adapted to a pressing safety problem, which matters as perioperative teams face growing workforce pressure and the operating room continues to digitalize. Such a system is not intended to replace the scrub nurse but to work alongside her as an independent second check, with responsibility for the count remaining with the perioperative team. Beyond the count, the instrument table may prove one of the few practical sources of structured data from open surgery, provided its output is logged in standardized form and linked to outcomes. A prospective study of nurse and system working together, beginning in the central sterile supply department and then moving into the operating room, is the essential next step.

## Data Availability

The datasets generated and analysed during the current study, comprising the run-level recognition data and the analysis code, are available from the corresponding author on reasonable request.

## Abbreviations

AI: Artificial intelligence
CI: Confidence interval
CPD: Count-presence detection
CLAIM: Checklist for Artificial Intelligence in Medical Imaging
GEE: Generalized estimating equation
ICC: Intraclass correlation coefficient
RSI: Retained surgical item(s)
